# Proline-Rich Antimicrobial Peptides from Invertebrates

**DOI:** 10.3390/molecules29245864

**Published:** 2024-12-12

**Authors:** Sylwia Stączek, Magdalena Kunat-Budzyńska, Małgorzata Cytryńska, Agnieszka Zdybicka-Barabas

**Affiliations:** Department of Immunobiology, Institute of Biological Sciences, Faculty of Biology and Biotechnology, Maria Curie-Skłodowska University, Akademicka 19 St., 20-033 Lublin, Poland; sylwia.staczek@mail.umcs.pl (S.S.); magdalena.kunat-budzynska@mail.umcs.pl (M.K.-B.); malgorzata.cytrynska@mail.umcs.pl (M.C.)

**Keywords:** proline-rich antimicrobial peptides, mode of action, PrAMP synthesis, PrAMP uptake by bacterial cells, DnaK, drosocin, apidaecin, oncocin, penaeidin, lumbricin

## Abstract

Antimicrobial peptides (AMPs) constitute a large and diverse group of molecules with antibacterial, antifungal, antiviral, antiprotozoan, and anticancer activity. In animals, they are key components of innate immunity involved in fighting against various pathogens. Proline-rich (Pr) AMPs are characterized by a high content of proline (and arginine) residues that can be organized into Pro-Arg-Pro motifs. Such peptides have been described in many invertebrates (annelids, crustaceans, insects, mollusks) and some vertebrates (mammals). The main objective of this review is to present the diversity of invertebrate PrAMPs, which are associated with the presence of cysteine-rich domains or whey acidic protein domains in the molecular structure, in addition to the presence of characteristic proline-rich regions. Moreover, PrAMPs can target intracellular structures in bacteria, e.g., 70S ribosomes and/or heat shock protein DnaK, leading to the inhibition of protein synthesis and accumulation of misfolded polypeptides in the cell. This unique mechanism of action makes it difficult for pathogens to acquire resistance to this type of molecule. Invertebrate PrAMPs have become the basis for the development of new synthetic analogues effective in combating pathogens. Due to their great diversity, new highly active molecules are still being searched for among PrAMPs from invertebrates.

## 1. Introduction

Antimicrobial peptides (AMPs) constitute a large and diverse group of molecules with antibacterial, antifungal, antiviral, antiprotozoan, and anticancer activity. In animals, they are key components of innate immunity involved in fighting against various pathogens. However, as multifunctional molecules, in addition to their antimicrobial properties, AMPs can play other important physiological roles in (i) immunomodulation, (ii) anti-inflammatory response, (iii) the control of gut homeostasis and microbiota, (iv) neuronal health, communication, and aging, and (v) the regulation of sleep and behavior [1]. Naturally occurring AMPs are usually cationic amphipathic molecules with a molecular weight of 3–10 kDa. In addition to these two common features, which play an essential role in the interaction with pathogen cell membranes, AMPs are highly diverse in terms of both molecular structure and the mechanism of their antimicrobial action. Taking into account the structure of the molecule, AMPs can be classified into three subgroups: (i) linear peptides without cysteine residues, able to form α-helices, (ii) peptides whose spatial structure is stabilized by disulfide bridges, and (iii) peptides with an overrepresentation of one of the amino acids in the sequence, e.g., glycine, tryptophan, or proline [1,2]. It is well documented that the microbial cell membrane is the main target of many AMPs [3,4,5]. Interactions with membrane phospholipids can result in the formation of (i) micelles leading to subsequent membrane fragmentation, (ii) pores (e.g., barrel-stave pores, toroidal pores), (iii) aggregates, (iv) non-bilayer intermediates, and (v) clusters of charged phospholipids. Other known consequences of such interactions include local thinning/thickening of the membrane or oxidized lipid targeting. These interactions lead to the depolarization and destabilization of the membrane, resulting in microbial cell death [6,7].

However, among naturally occurring AMPs, there is a group of peptides targeting intracellular structures. Their penetration into the microbial cell requires appropriate transporters located in the cell membrane. This group is represented by proline-rich AMPs (PrAMPs). In general, these peptides are characterized by a high content of proline (and arginine) residues. In natural PrAMPs, proline residues constitute 14–49% of the sequence. In addition, some PrAMPs contain one to several Pro-Arg-Pro (PRP) motifs [8]. PrAMPs have been described in many invertebrates (insects, crustaceans, mollusks) and some vertebrates (mammals), but not in primates or humans. Also, one PrAMP, named BnPRP1, has been characterized in the plant species *Brassica napus* [9]. Welch et al. [8] performed a broad analysis of the sequences and properties of known PrAMPs and those collected in the Data Repository of Antimicrobial Peptides (DRAMP) database [10] and proposed new, better-defined criteria for proline-rich AMPs. As proposed by the researchers, in order for a peptide to be classified as a PrAMP, it should meet the following criteria: (i) proline content not less than 25%, although the presence of the PRP motif is not essential, (ii) antimicrobial activity, (iii) intracellular targeting of DnaK or/and the 70S ribosome, and (iv) net charge at least +1. Some peptides classified so far as PrAMPs do not meet these criteria, whereas some others present in the DRAMP database could be new members of the PrAMP group. Nevertheless, recently reported research conducted using the genome mining approach resulted in the identification of 71 PrAMPs encoded in insect genomes [11], a new family of proline-rich cathelicidins (named rumicidins) in ruminants [12], and several new PrAMPs in cetaceans [13,14]. Given the ongoing development of research techniques, it is very likely that more peptides will be discovered, and their properties will be experimentally verified in the near future. This may eventually contribute to proposing further changes to the criteria used in the classification of PrAMPs, which has already been postulated by Handley et al. [15].

**Figure 1:**
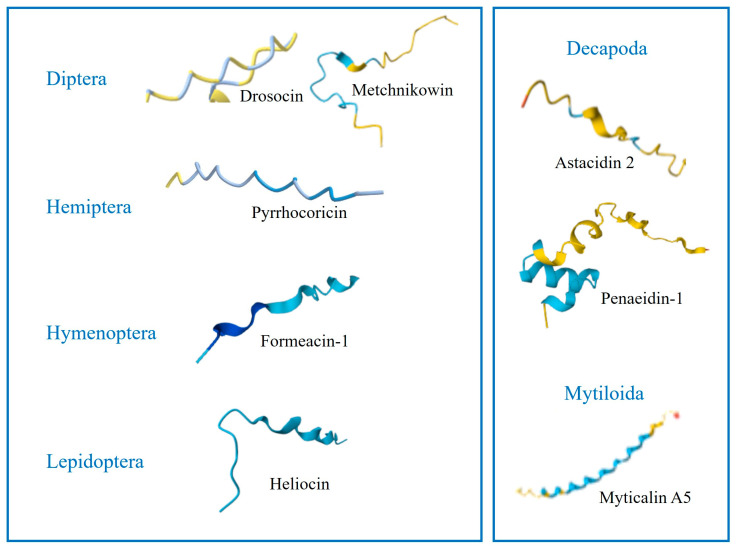
Examples of structures of mature natural PrAMPs from different invertebrates. The structures were generated using the AlphaFold Protein Structure Database based on the amino acid sequences of the peptides [16].

In this review, we present PrAMPs characterized in various invertebrates. The aim is to show the diversity of these invertebrate peptides; therefore, the work includes both peptides that fully meet the above-mentioned criteria and those whose structures contain a proline-rich domain in addition to other types of domains (Table 1, Figure 1). Furthermore, the work presents the mechanisms of the antimicrobial action of PrAMPs and the possibilities of using natural PrAMPs to develop more effective therapeutics.

## 2. Characteristics of Selected Invertebrate PrAMPs

### 2.1. Annelid PrAMPs

In annelids, antimicrobial peptides called lumbricins are relatively rich in Pro residues. They are usually 57–76 amino acids in length, and Pro residues comprise approximately 15% of the whole sequence. In addition, lumbricins contain multiple aromatic amino acid residues, including Phe, Tyr, or Trp (12–14% of the whole sequence), that can increase the spectrum of antimicrobial activity. The first one, lumbricin (lumbricin-1), was described in earthworms *Lumbricus rubellus*. A mature peptide is composed of 62 amino acids (7.2 kDa), among which Pro residues constitute 15%. Lumbricin-1 has a broad antimicrobial spectrum against fungi (*Candida albicans*, *Cryptococcus neoformans*, *Saccharomyces cerevisiae*), Gram-positive bacteria (*Bacillus subtilis*, *Staphylococcus aureus*, *Streptococcus mutans*), and Gram-negative bacteria (*Escherichia coli*, *Pseudomonas putida*, *Serratia* sp.) [17]. Lumbricin homologs were also described from earthworms *Pheretima tschiliens* [18]. A lumbricin-like AMP named lumbricin-PG, which shares 66% sequence identity with *L. rubellus* lumbricin-1, was isolated from the skin secretions of earthworms *Pheretima guillelmi*. Mature lumbricin-PG is composed of 59 amino acid residues, including 9 Pro residues (15.25% of the whole sequence). Lumbricin-PG has a broad antimicrobial effect, targeting a wide range of microorganisms from bacteria to fungi, with *P. aeruginosa* and *S. aureus* as the most sensitive strains [19]. More recently, two lumbricin homologs, Lumbr and lumbricin-related peptide (Lu-RP), have been identified in earthworms *Eisenia andrei*. Lumbr and Lu-RP are 63-amino-acid- and 59-amino-acid-long peptides (7.4 kDa, pI 7.95 and 7 kDa, pI 6.07), respectively. Their precursors show 66% identity [20].

### 2.2. Crustacean PrAMPs

The first AMP characterized in crustaceans was a 6.5 kDa PrAMP isolated from the hemolymph of the shore crab *Carcinus maenas*, showing similarity to mammalian bactenecin 7 (Bac7) [21]. Similar peptides were also identified in other species of mud crabs, such as *Scylla paramamosain*, *Scylla serrata*, and *Portunus pelagicus*, e.g., SpPR-AMP1 with activity against Gram-positive bacteria *Micrococcus luteus* and Gram-negative bacteria *Vibrio harveyi* [22].

Penaeidins comprise a family of peptides restricted to penaeid shrimps. The first penaeidins were isolated from the hemolymph of the Pacific white shrimp *Penaeus vannamei* [23]. These peptides can be divided into five subgroups (PEN1/2-PEN5) based on sequence similarities. Penaeidins contain two domains: the N-terminal proline-rich domain (PRD) and the C-terminal cysteine-rich domain (CRD) with six cysteines forming three disulfide bridges. PRD forms an extended structure, whereas the folded structure of CRD contains an α-helix stabilized by disulfide bonds [24,25,26]. Interestingly, the *PEN3* gene is widely distributed among penaeid shrimps, while the *PEN1/2*, *PEN4*, and *PEN5* genes are restricted to specific shrimp species. The diversification of penaeidins is thought to be due to gene duplication and positive Darwinian selection (adaptive evolution particularly in PRD domains), which have accelerated the rate of amino acid substitutions among these duplicated genes [27,28]. Penaeidins are primarily active against Gram-positive bacteria and filamentous fungi. A study on the PRD function in penaeidins revealed that this domain from PEN3 was devoid of antimicrobial activity, while PRD from PEN4 exhibited antibacterial and antifungal activity [29]. In addition, penaeidins can also act as cytokines. For example, *Penaeus monodon* penaeidin promoted the adhesion of shrimp granulocytes mediated by β-integrin [30]. In kuruma shrimp *Marsupenaeus japonicus*, a penaeidin (MjPen-II) containing a unique serine-rich region and typical PRD and CRD domains was described. It was shown that MjPen-II can bind to bacterial polysaccharides, thereby promoting bacterial agglutination. The serine-rich region and, to a lesser extent, the proline-rich domain were important for the agglutination activity of MjPen-II. It was also found that the peptide is involved in the phagocytosis of bacteria [31].

Another family of peptides with N-terminal proline-rich and C-terminal cysteine-rich domains comprises stylicins from the Pacific blue shrimp *Litopenaeus stylirostris*. The mature peptide (Ls-Stylicin1) contains 82 amino acids and is negatively charged with a theoretical pI of 5.0. The N-terminal domain contains 16 Pro residues (19.5% of the whole sequence), and the C-terminal domain contains 13 Cys residues (15.9% of the whole sequence). Using recombinant Ls-Stylicin1, it was demonstrated that the peptide strongly interacts with lipopolysaccharide (LPS), especially from *Vibrio penaeicidae*, and has high antifungal activity against *Fusarium oxysporum* [32].

Crustins are antimicrobial peptides (6–22 kDa, pI 4–8) that contain a whey acidic protein (WAP) domain and are widely distributed in crustaceans [33]. They are classified into four types (Type I–IV), depending on the sequence of the N-terminal domain. Among them, Type III crustins contain a proline-rich N-terminal region and exhibit activity against Gram-positive and Gram-negative bacteria. Type III crustins lacking this domain have been shown to lose their antibacterial activity, indicating an important role of the PRD domain in the antimicrobial action of these peptides [29].

Astacidins (Ast) comprise a family of short PrAMPs identified in crayfish. Astacidin was first identified in the plasma of the signal crayfish *Pacifastacus leniusculus* [34]. Four astacidins were also identified from the transcriptome of the red swamp crayfish *Procambarus clarkii*. The mature peptides are 20–31-amino-acid-long molecules. Their sequence alignment revealed that a central proline-rich motif in the PcAst-2 sequence has similarity to the corresponding motif in mammalian PrAMPs (Bac7, Tur1, PR-39) and in insect oncocin. Some astacins were demonstrated to be highly active against the Gram-negative bacteria *E. coli* and *Acinetobacter baumanii* [35].

**Figure 2:**
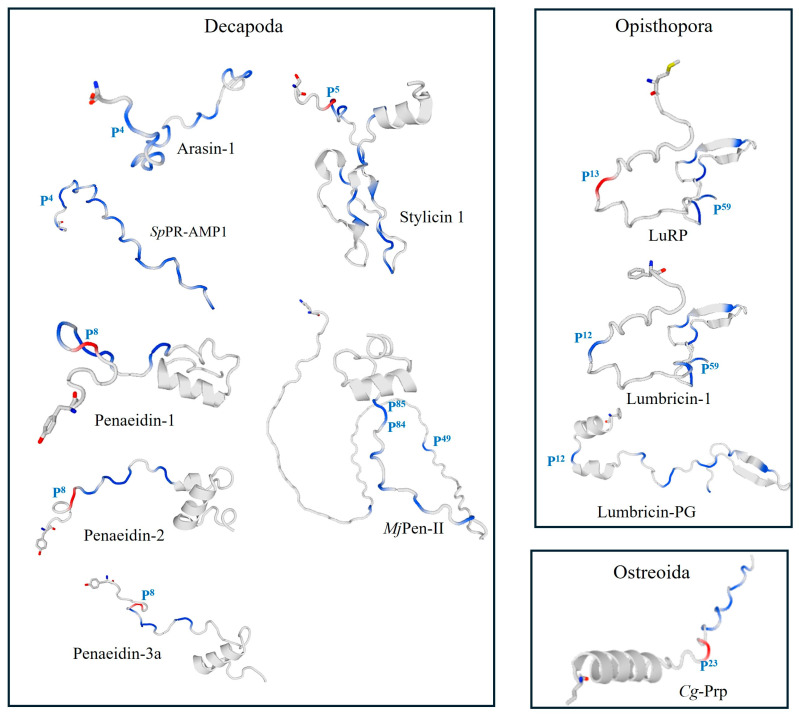
Examples of structures of mature natural PrAMPs from crustaceans, mollusks, and annelids. Proline residues are marked in blue and red. The first Pro residue in the Proline-rich region is labeled according to its position number in the sequence (e.g., P^4^). The N-terminal amino acid is indicated as a stick model. The structures were generated using the protein structure homology-modeling server SWISS-MODEL (https://swissmodel.expasy.org/ (accessed on 28 October 2024)) based on the amino acid sequences of the peptides [44].

In some crustaceans, e.g., the spider crab *Hyas araneus* [36], the crayfish *P. clarkii* [37], the mud crab *S. serrata* [38], and the shrimp *Litopanaeus vannamei* [39], arasin-like peptides have been characterized. Arasin 1 (37 aa) from *H. araneus* is composed of two regions: an N-terminal proline-rich region and a C-terminal stabilized with two disulfide bridges [36]. Arasin 1 exhibited activity against Gram-negative bacteria (*E. coli*, *Pseudomonas aeruginosa*, *Corynebacterium glutamicum*) and fungi (*S. cerevisiae*, *C. albicans*, *Botrytis cinerea*) [40] (Table 2, Figure 2).

**Table 2:** Properties of selected natural PrAMPs from crustaceans, mollusks, and annelids. Pro-Arg-Pro (PRP) motifs are indicated in blue, and Pro residues outside the PRP motifs are marked in bold. The first two N-terminal amino acids in the sequence of the *C. maenas* antibacterial 6.5 kDa peptide have not been identified and they are indicated as XX.

Order	Species	Peptide Name	Sequence	aa	% P	Ref
Decapoda	*Carcinus* *maenas*	Antibacterial 6.5 kDa peptide	XXV**P**YPRPFPRP**P**IGPRPL**P**F**P**GGGR**P**FQS	30	37	[21]
	*Hyas* *araneus*	Arasin-1	SRW**P**S**P**GRPRPFPGR**P**K**P**IFRPRPCNCYA**PP**C**P**CDRW	37	32	[36]
	*Marsupenaeus japonicus*	*Mj*Pen-II	KGSSSSSSSSRSSSSSYRSSGSSYRS**P**GSSYRSSGSYGTSGSRLSGIR**P**SSRSYRTGFRTAGSVG**P**ATR**P**FTR**P**TG**P**LK**P**ISR**PP**SRAACYSCYSASSATAIQCCTHYSLCCNLVKG	117	8	[31]
	*Pacifastacus leniusculus*	Astacidin 2	SLGYRPRPNYRPRPIYR**P**GK	20	25	[34]
	*Penaeus vannamei*	Stylicin 1	SSFS**PP**RG**PP**GWKL**P**CV**P**QEC**PP**C**P**YDDEC**P**KCGGF**P**VCHEVCTDISISCECGYHSCECPRPVCE**P**CES**P**IAELIKKGGYKG	82	18	[41]
		Penaeidin-1	YRGGYTG**P**IPRP**PP**IGR**PP**LRLVVCACYRLSVSDARNCCIKFGSCCHLVK	50	14	[23]
	*Scylla paramamosain*	*Sp*PR-AMP1	GYF**P**GR**PP**FPRPFPRP**P**SR**P**FPRP**P**F**P**G**P**FPRPY**P**WR	37	46	[22]
Mytiloida	*Mytilus galloprovincialis*	Myticalin A5	YSW**P**RM**P**RI**P**RL**P**RY**P**RY**P**RY**P**RW**P**RW**P**RQ**P**TIYA-NH2	35	29	[42]
Ostreoida	*Crassostrea* *gigas*	*Cg*-Prp	ILENLLARSTNEDREGSIFDTG**P**IRR**P**KPRPRPRPEG	37	16	[43]
Opisthopora	*Eisenia* *andrei*	LuRP	MYSKYERQKDKR**P**YSERKNQYTGQFLY**PP**ERI**PP**QKVIKWNEEGL**P**IYEI**P**GEGGHAE**P**AAA	62	13	[20]
	*Lumbricus rubbellus*	Lumbricin-1	FSKYERQKDKR**P**YSERKNQYTG**P**QFLY**PP**ERI**PP**QKVIKWNEEGL**P**IYEI**P**GEGGHAEPAAA	62	13	[17]
	*Pheretima guillelmi*	Lumbricin-PG	FSRYARMRDSR**P**WSDRKNNYSG**P**QFTY**PP**EKA**PP**EKLIKWNNEGS**P**IFEM**P**AEGGHIE**P**	59	15	[19]

### 2.3. Insect PrAMPs

#### 2.3.1. Dipteran PrAMPs

Drosocin (Dro), a 19-amino-acid AMP of *Drosophila melanogaster*, was described by Bulet et al. [45]. It contains three repeats of the PRP motif and a threonine residue (Thr11), which is O-glycosylated by the attachment of a monosaccharide N-acetylgalactosamine (α-D-GalNAc) or a disaccharide containing galactose linked to α-D-GalNAc (β-Gal(1→3)-α-D-64GalNAc). Both the mono- and disaccharide forms of Dro appear in *D. melanogaster* hemolymph within 6 h of infection. Glycosylation affects the antibacterial activity of the peptide. The activity of non-glycosylated Dro was 5–10 times lower than that of the glycosylated peptide, indicating that this post-translational modification is necessary for full activity [45,46]. It was reported that Dro lacking the C-terminal Arg18–Val19 almost completely lost antimicrobial activity [47]. Moreover, it has recently been demonstrated that mutations of Lys2, Pro16, and any mutation of the Arg residues of Dro diminish its activity, indicating their importance for Dro action [48,49].

Another PrAMP characterized in *D. melanogaster* is the 26-amino-acid peptide metchnikowin (Mtk). It is synthesized in the fat body as a 52-amino-acid prepropeptide upon microbial challenge and has similarities with the C-terminal sequence of honeybee abaecin [50]. Metchnikowin exhibits antifungal activity and acts against some phytopathogenic Ascomycota, such as *Fusarium graminearum* and *Blumeria graminis*; however, it is inactive against beneficial endophytic Basidiomycota, such as *Piriformospora indica* [51,52]. It was demonstrated that Mtk specifically targets the β(1,3)-glucosyltransferase and mitochondrial succinate–coenzyme Q reductase in sensitive fungi, causing perturbations in cell wall biosynthesis and a decrease in energy generation [52,53]. This ability of Mtk was exploited in transgenic barley, in which Mtk expression improved resistance against phytopathogenic Ascomycota fungi [51,54]. Mtk also has activity against the filamentous fungus *Neurospora crassa*, antiprotozoan activity against the malaria-causing parasite *Plasmodium falciparum* [55], and antibacterial activity against the Gram-positive bacteria *Micrococcus luteus* [50] and *Bacillus subtilis* [56]. Interestingly, it is inactive against the Gram-negative bacterium *E. coli* D31 at concentrations up to 5 µM [50]. However, the anti-*E. coli* DH5α activity of recombinant Mtk, obtained as a secretory peptide using an *E. coli* BL21 expression system, was detected by an agar plate diffusion assay (concentration approximately 8.2 µM) [56]. Considering the minimal inhibitory concentrations (MICs) of Mtk against fungi and Gram-positive bacteria in the range of 0.5–1.0 µM, the activity of this peptide against Gram-negative bacteria is very low. It is possible that in Gram-negative bacteria the inner membrane transporters responsible for the cellular uptake of some PrAMPs (e.g., Dro) are unable to transport Mtk due to structural differences. Hence, the two *D. melanogaster* PrAMPs, Dro and Mtk, appear to play distinct roles, being anti-Gram-negative bacteria and antifungal peptides, respectively. In addition to its involvement in innate immunity, a role of Mtk in the fly nervous system was suggested, as its engagement in the acute and chronic effects of traumatic brain injury caused by neuroinflammation was reported [57].

In the common green bottle fly *Lucilia sericata*, known for being used in maggot therapy for the efficient removal of necrotic tissues and the stimulation of healing of hard-to-heal wounds, four PrAMPs, named Lser-proline-rich peptide 1 (Lser-PRP1) (pI 12.0, 4.3 kDa), Lser-PRP2 (pI 12.2, 4.4 kDa), Lser-PRP3 (pI 12.2, 3.9 kDa), and Lser-PRP4 (pI 10.0, 2.8 kDa), were identified based on transcriptomic analysis. They share some sequence similarities with *Drosophila* Dro and Mtk. Lser-PRPs were inactive against *E. coli* and *M. luteus* at concentrations up to 100 µM [58,59]. Although neither Lser-PRP2 nor Lser-PRP3 was able to permeabilize the *E. coli* membrane, they exhibited anti-*E. coli* activity in the presence of stomoxyn, an α-helical AMP [60].

An interesting approach to preventing bacterial resistance may be the development of complex preparations based on natural insect AMPs, including PrAMPs. Such complex preparations containing different AMPs could be more effective than antibiotics. One of these is the complex of AMPs from dipteran *Calliphora vicina* larvae in which defensins, proline-rich peptides, cecropins, diptericins, and antiviral peptides (alleferons) were identified [61,62]. This preparation was active against bacteria from the families *Coccaceae*, *Corynebacteriaceae*, *Enterobacteriaceae*, *Enterococcaceae*, *Moraxellaceae*, and *Pseudomonadaceae*. Chernysh et al. [62] investigated differences in the development of bacterial resistance as a result of treatment with a natural complex of *C. vicina* AMPs and reference antibiotics, including β-lactams and polymyxin B. The tested bacteria (*E. coli*, *A. baumanni*, and *Klebsiella pneumoniae*) very quickly developed resistance to the individual antibiotics, but they were unable to develop resistance to the complex of *C. vicina* AMPs. Similar observations have been made regarding the effect of AMP complexes obtained from other dipteran species, i.e., *Calliphora vomitoria*, *Lucilia sericata*, and *Musca domestica* [62,63] (Table 3).

#### 2.3.2. Hemipteran PrAMPs

Pyrrhocoricin was purified from the hemolymph of the firebug *Pyrrhocoris apterus* immunized with *M. luteus* and *E. coli* [65]. In this 20-amino-acid peptide (pI 10.3, 2.54 kDa), Pro residues constitute 25% and the sequence contains two PRP motifs typical for PrAMPs. Similarly to Dro, pyrrhocoricin is O-glycosylated at Thr11 by the attachment of the Gal-GalNAc disaccharide (Table 3). Interestingly, at different concentrations ranging from nanomolar to 10 µM, pyrrhocoricin inhibited the growth of Gram-negative bacteria (*E. coli*, *P. aeruginosa*, *Salmonella Typhimurium*, *K. pneumoniae*, *Enterobacter cloaceae*, *Agrobacterium tumefaciens*), as well as some Gram-positive bacteria (*M. luteus* and *Bacillus megaterium*) [65,74,75].

Other PrAMPs, named metalnikowins, have been described in the green shield bug *Palomena prasina*. There are four forms of metalnikowins containing 15–16 amino acids and five (metalnikowin-1, -2A, -3) or four (metalnikowin-2B) Pro residues. These peptides are not post-translationally modified and appear to be bacteriostatic, exhibiting low activity against Gram-negative bacteria [66,75].

Oncocin represents a class of PrAMP analogs of the antibacterial peptide originally isolated from the milkweed bug *Oncopeltus fasciatus*. The original peptide, named Oncopeltus peptide 4, was obtained from the hemolymph of *O. fasciatus* infected with *P. aeruginosa* and *P. putida*. It contains 20 amino acids (VDKPPYLPRPPPPRRIYNNR), with a high proportion of Pro residues (30%) and a large number of cationic amino acids (25%). Peptide 4 exhibits 77.8% identity with *P. apterus* pyrrhocoricin and 76.9% identity with *P. prasina* metalnikowin-1 [67]. Many analogs have been developed based on the sequence of Oncopeltus peptide 4 in order to increase the antimicrobial activity spectrum. Peptide 4 was only very slightly active against *E. coli* and *M. luteus*, while one of its analogs with three introduced changes, i.e., the replacement of Pro11 with Arg, the deletion of one Asn residue at the C-terminus, and the addition of C-terminal amidation (VDKPPYLPRPRPPRRIYNR-NH2), was highly active against different Gram-negative bacteria [76]. Oncocin112, in which Arg15 and Arg19 are substituted with D-Arg, has been extensively studied (see Section 5.2), as has its mechanism of antibacterial action [77,78,79].

#### 2.3.3. Hymenopteran PrAMPs

Apidaecins (Api), a family of short 18-amino-acid PrAMPs, were first discovered in *Apis mellifera* [80]. Three different isoforms were isolated from the hemolymph of honeybees immunized with *E. coli* and characterized, namely Api-1A (Val6, Ile18), Api-1B (Val6, Leu18), and Api-2 (Ile6, Leu18), differing in amino acid residues at positions 6 and 18. In addition, based on the cDNA sequence, a fourth apidaecin isoform (Api-3) with Ser instead of Pro at position 9 was deduced; however, this isoform was not detected in the hemolymph. It was reported that Api-1B is the most abundant isoform in the honeybee hemolymph. This may be due to the fact that apidaecins are synthesized as three precursor polypeptides, i.e., apidaecin type 73, apidaecin type 22, and apidaecin type 14, encoded by *Apid73*, *Apid22*, and *Apid14*, respectively. These precursors contain, in total, 11, 3, 2, and 2 repeats of the Api-1B, Api-1A, Api-2, and Api-3 sequences, respectively [69,81,82]. Apidaecin-like peptides were also characterized in other hymenopteran species, mainly bees and wasps. These PrAMPs are predominantly active against Gram-negative bacteria at nanomolar doses [11,70,81]. Apidaecins are non-lytic AMPs inhibiting bacterial protein synthesis by binding to the ribosome and trapping release factors associated with the terminating ribosome [83]. Moreover, another mechanism of action against Gram-negative bacteria was proposed based on the results of studies using the iTRAQ-coupled 2-D LC-MS/MS technique by Zhou and Chen [84,85]. It was shown that the action of Api-1B on *E. coli* cells caused a decrease in the production of GroEL/GroES chaperonins and also resulted in an unbalanced synthesis of LPS and membrane phospholipids via an increase in the production of ftsH protease and the associated increased degradation of UDP-3-O-acyl-N-acetylglucosamine deacetylase involved in the biosynthesis of the lipid A moiety of LPS [84,85].

Abaecins are other PrAMPs described in hymenopteran species. Abaecin was first characterized in *A. mellifera* as a 34-amino-acid peptide that contains 10 Pro residues (29% of the whole sequence) (Table 3). Pro residues are distributed over the entire length of abaecin, thus excluding the possibility that the peptide adopts an α-helical conformation. Abaecins are active against both Gram-negative and Gram-positive bacteria. However, in comparison to apidaecin, abaecin is generally 200 times less active against *Agrobacterium*, *Erwinia*, and *E. coli* strains [70,81].

**Figure 3:**
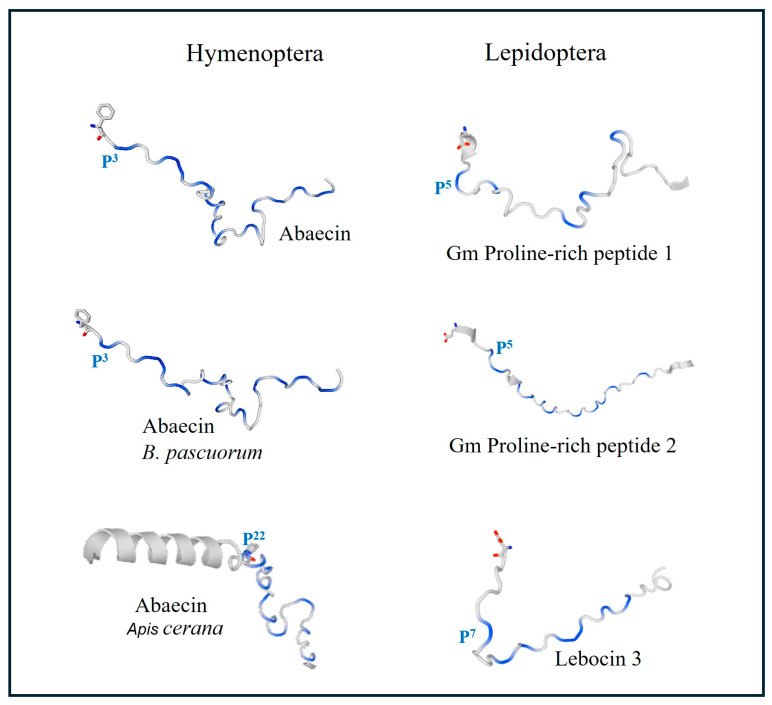
Examples of structures of mature natural PrAMPs from two insect orders, Hymenoptera and Lepidoptera. Proline residues are marked in blue. The first Pro residue in the Proline-rich region is labeled according to its position number in the sequence (e.g., P^5^). The N-terminal amino acid is indicated as a stick model. The structures were generated using the protein structure homology-modeling server SWISS-MODEL (https://swissmodel.expasy.org/ (accessed on 28 October 2024)) based on the amino acid sequences of the peptides [44].

In the bulldog ant *Myrmecia gulosa*, two 16-amino-acid PrAMPs called formaecins 1 and 2 (F1 and F2) were characterized by Mackintosh et al. [71]. The primary structure of F1 and F2 differs at positions 8 and 13. Proline residues comprise approximately 30% of the formaecin sequence. Interestingly, the PRP motif is replaced in formaecins by PNP and PHP sequences. Similarly to drosocin and pyrrhocoricin, formaecins contain O-glycosylated Thr11. Both formaecins are heat-stable and cationic peptides. The theoretical pI of F1 is 12.0 and that of F2 is 11.0; at physiological pH, they have a net charge of (+4) and (+3), respectively. F1 and F2 have 50% and 44% sequence identity with drosocin, respectively. Both peptides exhibited activity against *E. coli*, but had no effect against Gram-positive bacteria, yeast *C. albicans*, eukaryotic cells, or pestiviruses [71,74] (Table 3, Figure 3).

#### 2.3.4. Lepidopteran PrAMPs

The first PrAMP found in a lepidopteran was a 32-amino-acid lebocin isolated by Hara and Yamakawa [86] from the hemolymph of a *Bombyx mori* silkworm immunized with *E. coli*. The authors demonstrated that Thr15 was modified by O-glycosylation and the peptide sequence was 41% similar to *A. mellifera* abaecin. Three lebocin isoforms were identified in *B. mori* (Table 3). Lebocins 1 and 2 have the same sequence but are glycosylated by different sugars: lebocin 1 by Gal-GalNAc and lebocin 2 by GalNAc. In turn, in lebocin 3, Thr15 is modified with GalNAc; however, this isoform contains Leu16 instead of Pro16, which is present in lebocin 1 and 2. The amino acid sequence of another isoform, lebocin 4, was deduced on the basis of cDNA analysis [72,87]. Lebocin has antibacterial activity against *Acinetobacter* sp. and *E. coli* and can act in cooperation with *B. mori* α-helical cecropin D. In the presence of lebocin, the increased permeability of liposomal membranes in low-ionic-strength conditions was observed, which suggested that it can disturb the bacterial membrane [86]. Lebocins are Lepidoptera-specific PrAMPs. They are processed from precursor polypeptides that have been identified in many lepidopteran species, including *Manduca sexta*, *Pseudoplusia includens*, *Samia cynthia*, *Spodoptera litura*, and *Trichoplusia ni*. Lebocin genes are mainly expressed in the fat body after infection and, to some extent, in hemocytes [74,88,89,90,91,92,93,94].

Two unique PrAMPs, named proline-rich peptide 1 and 2 (Pro1 and Pro2), were identified in the greater wax moth *Galleria mellonella* (Gm), a model organism widely used in research on the mechanisms of insect innate immunity and the pathogenicity of various microorganisms, including human pathogens. GmPro1 contains 37 amino acids (pI 11, 4.3 kDa), including five Pro residues, whereas GmPro2 is composed of 42 amino acids (pI 9.97, 4.9 kDa) and contains eleven Pro residues. Proline constitutes 13.5% and 26.2% of the GmPro1 and GmPro2 sequences, respectively. Both peptides lack typical PRP motifs but contain the KP and PR motifs observed in long-chain PrAMPs [73,95] (Table 3, Figure 3). Brown et al. [96] showed that both *G. mellonella* PrAMPs are encoded in one gene together with anionic peptide 1 and a heliocin-like peptide, suggesting that mature forms of these AMPs are processed from a precursor polypeptide [96]. It was reported that GmPro1 was active primarily against yeast and yeast-like fungi. In addition, both peptides had low activity against *M. luteus*. However, none of the peptides showed activity against *E. coli* [73], but further studies demonstrated that GmPro2 purified from hemolymph inhibited the growth of the entomopathogenic bacterium *Pseudomonas entomophila* [97].

#### 2.3.5. Insect PrAMPs—Short Evolutionary Perspective

In light of studies showing the large diversity of invertebrate PrAMPs, a question arises about the causes and origins of this diversity. In general, many AMPs act in a non-specific manner, but having different structures and biochemical properties and acting as complex mixtures, they enable the immune systems of invertebrates to combat a variety of pathogens and parasites more effectively. However, there is evidence for a hypothesis that AMP diversity may be due to highly specific interactions between some AMPs and subsets of pathogens targeted by these AMPs. Using single and multiple knockouts and different bacterial and fungal pathogens, the in vivo function of *D. melanogaster* AMPs was analyzed by Hanson et al. [98]. It was revealed that AMPs, including PrAMPs, can play highly specific roles in defense against certain pathogens. For example, drosocin has been shown to be specifically required for defense against *Enterobacter cloacae*; diptericins alone are critical for resistance against *Providencia rettgeri* (a Gram-negative bacterium and a natural pathogen of *Drosophila*); and metchnikowin, together with drosomycin, contributes to defense against yeast *Candida albicans*. Similarly, in their research on the diversity of apidaecin-like peptides, Huang et al. [99] found that the diversity of Api homologs in bees and wasps reflects the adaptability of these PrAMPs in different insect species to their role in innate immunity, which is likely driven by the spectrum of microbial pathogens able to infect the insect host [11]. These studies indicated that AMPs experience a positive selection, driven by the evolutionary race of host–pathogen interactions [98,100,101]. In their study on Diptera AMPs, Hanson et al. [102] showed the independent loss of some AMPs in different lineages of this insect order as a result of, e.g., pseudogenization or segregating null alleles. It was found that, in dipteran species living in a more sterile environment, there was a loss of some AMP families. Two PrAMPs, namely drosocin and metchnikowin, are restricted to the Drosophila genus and their close relatives. Interestingly, an antifungal metchnikowin was recovered from diverse mushroom-feeding Drosophila. This indicates that selection in the innate immune system can act directly on AMPs, suggesting that, in the absence of a microbial challenge, some AMPs are not necessary and even deleterious to fitness [102,103,104].

### 2.4. Mollusk PrAMPs

The first PrAMP from mollusks was described in the oyster *Crassostrea gigas* and was identified by screening a hemocyte EST library for PRP motifs. Based on cDNA analysis, the deduced amino acid sequence revealed the presence of a 61-amino-acid-long polypeptide precursor, which contained a mature 37-amino-acid peptide named *Cg*-Prp. Interestingly, this 4.24 kDa peptide with a pI of 10.53 contained a proline-rich region at the C-terminus. The amino acid sequence alignment revealed that this region contains two PRP motifs and is homologous to some insect PrAMPs, e.g., abaecin, drosocin, and metalnikowin [43].

Two families of AMPs, named myticalins and modiocalins, were identified by bioinformatics analyses of genomic and transcriptomic data from the marine mussels *Mytilus galloprovincialis* and *Modiolus philippinarum*, respectively [42]. Mature myticalins are 23–42-amino-acid peptides divided into four subfamilies (A to D), three of which (A, B, D) include peptides in which Pro residues constitute 21–28% of the sequence. Myticalins have a broad spectrum of activity against both Gram-positive (*S. aureus*, *B. subtilis*) and Gram-negative (*E. coli*, *P. aeruginosa*, *A. baumanii*) bacteria [42,105]. Multiple cationic PrAMPs were also isolated from the hemolymph of the marine snail *Rapana venosa*. In the N-terminal region, they contain two or three Pro residues, and this region has high homology with *Penaeus vannamei* and *Penaeus stylirostris* penaeidins [106].

## 3. PrAMP Synthesis and Uptake by Bacterial Cells

In general, PrAMPs are synthesized by ribosomes as inactive precursors with varied structure [107,108]. Some precursors contain a pre-sequence, a pro-sequence, and a single peptide sequence (e.g., insect drosocin, mammalian PrAMPs), whereas others, in addition to the pre- and pro-sequences, contain multiple copies of the peptide sequence separated by a conservative oligopeptide. Such multiple-copy precursors have been described for insect PrAMPs, e.g., apidaecin from *A. mellifera* (Hymenoptera), riptocin from *Riptortus pedestris* (Hemiptera), and proline-rich peptides 1 and 2 from *G. mellonella* (Lepidoptera) [69,96,109]. Recently reported new apidaecin-like PrAMPs identified in insect genomes (bees and wasps; Hymenoptera) are also encoded as polypeptides containing a varying number of PrAMP repeats separated by a conservative motif [11].

**Figure 4:**
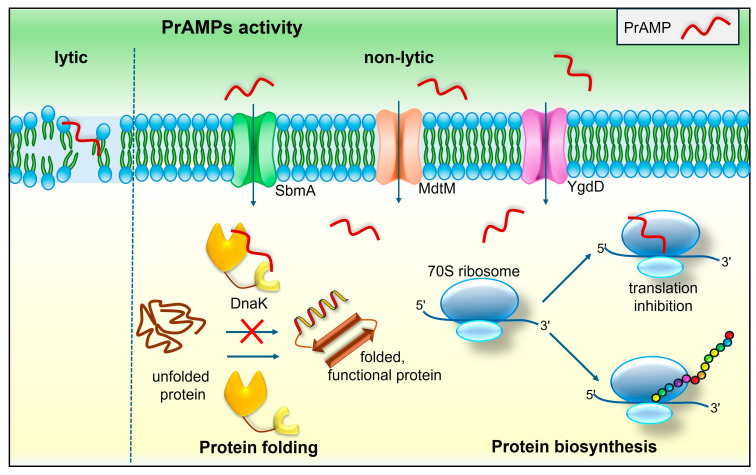
Mechanisms of action of PrAMPs. Non-lytic PrAMPs (**right**) are transported into the bacterial cell by the inner membrane proteins SmbA/BacA, MdtM, and/or YgdD. Once inside the cell, they bind to 70S ribosomes and/or DnaK chaperones, leading to the inhibition of protein synthesis and/or the improper folding of polypeptides. A lytic mode of action (**left**) involves peptide interaction with the membrane, causing its destabilization and damage, which results in cell death.

Insect and mammalian PrAMPs are primarily active against Gram-negative bacteria and target intracellular structures: the 70S ribosome and/or the DnaK protein. Unlike many other AMPs, which kill bacterial cells through the destabilization and disruption of bacterial membranes as a result of interactions with membrane phospholipids, PrAMPs are transported into bacterial cells by some inner membrane proteins and cause non-lytic bacterial death (Figure 4) [107,108]. The major transporter responsible for PrAMP uptake into bacterial cytosol is the SmbA protein present in many Gram-negative bacteria, e.g., *Enterobacteriaceae* (*E. coli*, *K. pneumoniae*, *Salmonella*) and *Pseudomonadales* (*A. baumanii,* but not in *P. aeruginosa*) [107,108,110,111]. Besides PrAMPs, the SmbA transporter is also engaged in the internalization of other structurally diverse antimicrobial agents. It shares some common features with ATP-binding cassette transporters (but does not contain a nucleotide-binding domain) and is a member of the peptide uptake permease family [112,113]. SmbA functions as a homodimer with 8 transmembrane α-helices per 45 kDa protomers, and transports peptide molecules using proton driving forces [110,112]. Recently, a molecular mechanism exploited by SmbA for AMP transport has been proposed by Ghilarov et al. [111]. The *Sinorhizobium melilotti* BacA transporter, closely related to SmbA and included in the family of peptide uptake permeases, has also been implicated in PrAMP transport [113,114]. MdtM and YgdD are other inner membrane proteins involved in PrAMP uptake. The MdtM protein is a drug/H+ antiporter belonging to the major facilitator superfamily (MFS) of multidrug resistance transporters involved in the efflux of, among others, antibiotics and biocides. Its role in PrAMP uptake was discovered in a study on alternative uptake mechanisms upon the finding that *E. coli* SmbA mutants were still susceptible to some PrAMPs [112]. The role of the YgdD protein in PrAMP uptake by *E. coli* cells was detected during the screening of a single-gene deletion mutant library against arasin 1(1–23) from the spider crab *H. araneus*. It was demonstrated that, for full *E. coli* susceptibility to this PrAMP, both inner membrane proteins SmbA and YgdD are necessary; however, it was not clear whether YgdD plays a role of a peptide transporter or rather facilitates peptide uptake [115]. Further research on the role of SmbA and YgdD in apidaecin-1B and pyrrhocoricin transport using *E. coli* individual knockouts of each transporter revealed that SmbA is the primary while YgdD is probably the secondary transporter of these two insect PrAMPs [116].

It is worth noting that other AMPs, capable of forming pores in membranes, may be helpful in the entry of some PrAMPs into the bacterial cell. Such cooperation has been described for the AMPs of the common green bottle fly *L. sericata* (Diptera), where α-helical stomoxyn enhanced the activity of PrAMP Lser-PRP2 against *E. coli* [60]. The functional interaction against *E. coli* between bumblebee PrAMP abaecin and pore-forming hymenoptaecin, cecropin A, and stomoxyn was also demonstrated in other studies [117,118].

## 4. Mechanisms of Antimicrobial Action of PrAMPs

Once inside a bacterial cell, intracellularly acting PrAMPs can disrupt ribosomal protein synthesis and/or chaperone DnaK-assisted protein folding thanks to the ability of interaction with 70S ribosomes and/or DnaK chaperones, respectively (Figure 4) [108].

Initial research on the intracellular targets of insect PrAMPs, i.e., apidaecin, drosocin, and pyrrhocoricin, indicated the DnaK chaperone as the main target of these peptides. The interaction of these peptides with DnaK can result in permanently closing the lid over the binding pocket in the DnaK molecule, preventing polypeptide binding to the chaperone. In addition, PrAMP binding can lead to inhibition of DnaK ATPase activity. Such interactions cause improper protein folding, defective ribosome biogenesis, and the accumulation of protein aggregates in the cell [74,119,120,121,122]. In addition, the study of the insect PrAMPs apidaecin and pyrrhocoricin showed that, in the case of non-lytic PrAMPs, D-enantiomers lose their antibacterial activity, unlike in the case of membrane-lytic AMPs. D-apidaecins bound with bacterial cells as fast as L-apidaecins, but unlike the L-counterparts, they were not internalized [123]. Similarly, L-pyrrhocoricin diminished the ATPase activity of recombinant DnaK and reduced the DnaK refolding function, while D-pyrrhocoricin was unable to inhibit DnaK [119,120]. Further research demonstrated that *E. coli* wild-type and *E. coli* DnaK deletion strains exhibited similar susceptibility to PrAMPs, suggesting that although PrAMPs can interact with DnaK, this is not a primary mechanism of their antibacterial activity [124,125,126,127]. However, there is evidence that at least for two PrAMPs, i.e., *L. sericata* Lser-PRP2 and Lser-PRP3, the interaction with the DnaK chaperone can play a crucial role in antibacterial action, as it was found that they do not inhibit translation [60]. Nevertheless, based on vast amounts of research, the inhibition of protein synthesis through binding to 70S ribosomes is considered the primary mechanism of action of many PrAMPs [11,48,49,107,108].

PrAMPs bind to prokaryotic ribosomes within the nascent polypeptide exit tunnel (NPET) in the 50S ribosomal subunit [108]. However, they differ in their mode of binding and mechanism of action. Class I PrAMPs, which include most of the PrAMPs tested in this respect (pyrrhocoricin, metalnikowin, oncocin, bactenecin7, Tur1A), bind with an inverted orientation compared to the nascent polypeptide and interfere with the A-site binding pocket and the A-site crevice. Such binding prevents the first step of translation elongation by impeding the delivery of the first aa-tRNA, resulting in ribosomal stalling at the AUG start codon. The proper binding of class I PrAMPs requires the presence of a conserved core containing the PRP motif [108]. On the other hand, class II PrAMPs (apidaecin and Api-like peptides, drosocin) bind to the NPET with a similar orientation to the nascent polypeptide, with the important residues located in the peptidyltransferase center (PTC). This binding takes place after releasing the nascent polypeptide from the ribosome and leads to trapping the release factors (RFs) and tRNAs in the PTC. In this way, the ribosome is arrested at the stop codon. RF sequestration on the terminating ribosomes by class II PrAMPs reduces the pool of free RFs in the cell and thus deprives the remaining ribosomes of the ability to release the synthesized polypeptides and properly terminate translation. It can also result in stop codon readthrough by the ribosomes and the synthesis of abnormal proteins with additional extensions at the C-termini [83,99,128]. It was demonstrated that the highly conserved C-terminal region of apidaecin and Api-like peptides is crucial for their activity [11,108]. However, the results reported by Mangano et al. [48] provide evidence that drosocin and apidaecin interact with the target differently. Although drosocin arrests ribosomes at stop codons and is able to sequester RF1, its interaction with the ribosome relies on many amino acid residues distributed throughout the molecule [48]. Moreover, an important role of drosocin posttranslational modification, i.e., O-glycosylation at Thr11, in the interaction with 23S rRNA was demonstrated by Koller et al. [49].

Since the invertebrate PrAMPs described so far differ in their amino acid sequences, spatial conformation, molecular length, and arrangement of hydrophobic and hydrophilic regions, diverse modes of their antimicrobial action can be assumed [29]. While the mechanisms of action of some insect PrAMPs (apidaecin, drosocin, metchnikowin, pyrrhocoricin, oncocin) have largely been elucidated, the mode of action of other invertebrate PrAMPs still remains to be explained. The question is whether they have a non-lytic or rather lytic mode of action.

A dual mode of action has been proposed for some PrAMPs and their derivatives, e.g., crustacean arasin 1 and mammalian bactenecin7 (Bac7). This duality seems to reflect the structure and length of the peptides. In the case of Bac7 (59 aa) and its truncated derivatives, it was demonstrated that higher membrane permeabilizing activity correlated with longer and more hydrophobic molecules [107,108]. *Hyas araneus* arasin 1 contains the N-terminal proline-rich and C-terminal region stabilized with two disulfide bridges [36]. It was revealed that the activity of the arasin 1(1–23) N-terminal fragment was similar to the full-length peptide. For arasin 1(1–23), the non-lytic mechanism was described at the MIC of the peptide, whereas at concentrations higher than MIC, the lytic mode was the primary mechanism of killing bacterial cells [40,115]. Myticalins characterized in marine mollusk *M. galloprovincialis* are another example of PrAMPs that exploit the lytic mechanism of bacterial killing, causing permeabilization of *E. coli* membranes. It is worth noting that the consensus amino acid sequence present in non-lytic PrAMPs and important for the interaction with 70S ribosomes is absent from myticalin sequences [42,105]. Another example is *Cg*-Prp from *C. gigas*. Although its mode of action was not characterized, it was shown that the full-length *Cg*-Prp as well as its C-terminal fragments exhibited very low antibacterial activity, which increased considerably in the presence of *C. gigas* defensin [43]. Given the homology to intracellularly active insect PrAMPs, one can speculate that defensin enables *Cg*-Prp and derivatives to enter the cell and reach their targets (Figure 4).

## 5. Synthetic Derivatives of Invertebrate PrAMPs

From the point of view of practical application possibilities, the essential features of a promising antimicrobial peptide include (i) selective toxicity towards pathogens while maintaining safety for host cells, (ii) the fast killing of pathogens, (iii) a broad activity spectrum, and (iv) a mechanism of action that makes it difficult or even impossible for pathogens to develop resistance. Research on the structure–activity relationships of different AMPs revealed net charge, hydrophobicity, and amphipathicity as the most important physicochemical and structural determinants of antimicrobial activity. AMP properties, including cytotoxicity and stability, can be influenced by alterations in these interrelated parameters. Especially, properly balanced charge, hydrophobicity, and imperfect amphipathicity have a positive impact on the effectiveness of AMPs. The development of novel, more effective and safe peptide molecules based on invertebrate PrAMPs requires the optimization of many factors to overcome potential obstacles, e.g., toxicity against host cells, hemolytic activity, instability in body fluids, susceptibility to proteolytic degradation, undesirable immunomodulatory activity, and the potential development of microbial resistance [129,130,131,132,133].

Based on natural PrAMPs, synthetic forms with greater antimicrobial activity and without a negative effect on host cells are being developed. The expected features of new peptides include (i) stability under protease action, (ii) a non-lytic mode of action, and (iii) involvement of the bacterial transporter system in cellular uptake, which allows peptides to act on intracellular targets, e.g., chaperone DnaK or 70S ribosomes. Synthetic peptides may also act synergistically with antibiotics, which may improve the efficacy and reduce the dosing frequency of these antibiotics [134,135,136]. 

### 5.1. Apidaecin-Derived Peptides

Based on the structure of apidaecin 1B (18 aa) and using multi-substitution libraries of genetically encoded and endogenously expressed variants, new peptides with functions similar to those of apidaecin were discovered, i.e., blocking translation by binding to the ribosome exit channel, where RF1 or RF2 are captured, resulting in ribosomes stopping at stop codons. By orthogonal strategies using positive and negative selection, new peptides targeting ribosomes and some with antibacterial properties were identified [99]. The essential elements of new Api-like peptides are the N-terminus and C-terminus of the amino acid sequence. The N-terminus is responsible for regulating the action and function of the peptides, while changes in the C-terminus can lead to negative effects, e.g., reduced antibacterial activity. However, it has been shown that some substitutions in the C-terminus of natural PrAMPs are possible, e.g., one or two substitutions occur in the new insect Api-like peptides Oab1 and Bvo1, identified by the genome mining approach in *Orussos abietinus* and *Bombus vosnesenski*, respectively, that do not reduce the antibacterial activity of the peptides [11,99].

Api137 and Api88 are derivatives of apidaecin 1B, from which they differ at positions 1 (Gly1Orn) and 10 (Gln10Arg) and in N-terminal guanidation. Both analogues differ in their C-termini. In Api88, the C-terminus is amidated, which removes the only negative charge of the peptide, making the peptide more cationic. The peptide is more resistant to the protease action, and therefore more stable, but this modification also results in higher cellular uptake rates [137,138]. Both peptides are characterized by higher antibacterial activity compared to the parent molecule. Intraperitoneal injections of Apo88 and Api137 to mice with fatal septicemia caused by pathogenic *E. coli* ATCC 25922 rescued these animals without symptoms of toxicity [137,139,140]. Further pharmacokinetics studies demonstrated that the continuous subcutaneous infusion of Api137 was more effective in treating intraperitoneal sepsis in mice, compared to other routes of administration [141], whereas a new non-rodent model for invasive *K. pneumoniae* bacteremia, i.e., ex vivo tests in fresh porcine blood, revealed an Api137 bactericidal effect against hypervirulent *K. pneumoniae* [142].

In addition to work on improving the developed Api analogs towards their potential medical use, research conducted on these Api-derived peptides provided further detailed information on the mechanism of the antibacterial action of insect PrAMPs. Api137 inhibits protein synthesis via trapping release factors (RFs). However, Lauer et al. [143] showed that the inhibitory effect of Api88 and the partial inhibitory effect of Api137 are based on an RF1-independent mechanism. Both Api137 and Api88 have been shown to inhibit bacterial ribosomes in multiple ways, having several different binding sites to the ribosome. Cryo-EM and MD data indicated that these peptides are electrostatically attracted to the negatively charged ribosome. At the NPET entrance, the positively charged peptide—mainly its Arg, His15, Tyr7, and Pro residues—interacts with nucleobases. Then, Api137 and Api88 migrate to the negatively charged interior of the NPET tunnel. The movement of Api137 stops earlier due to the negatively charged C-terminus, which hinders the passage through the narrow part between the negatively charged helices H51 and H49 of the 23S rRNA. On the other hand, due to its stronger positive charge, Api88 is able to move further towards the PTC. In addition, Api88 also has a third unique binding site in domain III of 23S rRNA [143]. Api88 and Api137 bind to NPET in the same orientation as the nascent polypeptide chain and use RxPP, PR repeats, and polyproline motifs. Interestingly, motifs of these types are rarely present in natural proteins because they act as self-inhibiting motifs of protein synthesis [143,144,145].

Api805, developed based on Api137, is another synthetic peptide which evidences that small structural alterations in PrAMP molecules change their binding sites and mechanism of action. It contains the N-terminal sequence of Api137, with a guOrn1Gly substitution, and the C-terminal Leu residue is replaced by four residues of the drosocin C-terminal sequence, i.e., Pro-Ile-Arg-Val. In addition, it contains a Val6Ile substitution [128]. Interestingly, Api805 has varying activity against different strains of *E. coli*. To explain these differences, analyses of the dissociation constants for 70S ribosomes, mutations in the SbmA transporter, and the possibility of the proteolytic degradation of Api805 by bacterial proteases were performed [135]. Due to the structural similarity of Api805 to apidaecin and drosocin, a similar binding site to the ribosome was expected as in Api137, although the C-terminus is modified and the part important for the Api137 interaction with the ribosomal P-site is shifted by three residues in Api805 [128]. Api805 bound to *E. coli* ribosomes as strongly as Api137 and more strongly than drosocin. However, Api805 weakly competed with Api137 for the binding sites in the ribosome. Similarly, Api88 strongly bound to the 70S ribosome but competed weakly with Api137 [112,146]. It was concluded that Api805 probably binds to different sites in the 70S ribosome compared to other PrAMPs and inhibits translation in an RF-independent manner, similar to Api88. While Api137 and drosocin share a similar binding site in the 70S ribosome and inhibit translation in an RF1-dependent manner, Api805 and Api88 reduce translation to a lesser extent and independently of RF1 [135].

### 5.2. Oncocin Derivatives

The OM19R peptide was developed based on fragments of oncocin and a *Musca domestica* AMP called MDAP-2 [147,148]. In comparison with oncocin and MDAP-2, OM19R showed better potency against the Gram-negative bacteria *E. coli, S. typhimurium, Shigella flexneri*, and *Shigella dysenteriae*. The transport of this peptide into bacterial cells was dependent on the SmbA transporter, and no membrane damage was observed [148]. Based on the OM19R peptide, a new PrAMP called OM19r was developed by substituting L-Arg with D-Arg at positions 15 and 19 [149]. The OM19r peptide showed high activity against many standard strains and clinical isolates of Gram-negative bacteria, including *E. coli*, *Salmonella, Shigella, Acinetobacter,* and *K. pneumoniae,* but was inactive against Gram-positive bacteria. In combination with the aminoglycoside antibiotic gentamicin, often used to treat infections caused by drug-resistant bacteria, OM19r caused a 64-fold increase in the gentamicin activity against multidrug-resistant (MDR) *E. coli* B2. It was also shown that OM19r was transported into bacterial cells by SbmA and increased the permeability of the inner membrane of *E. coli* B2, showing better efficacy than gentamicin. No effect of OM19r and gentamicin on the outer membrane of this bacterium was observed. It was revealed that the peptide and gentamicin interact with different sites in the 70S ribosome, which enhances the antibacterial effect, i.e., gentamicin inhibits the initiation of translation, while OM19r inhibits the elongation process. Interestingly, a synergistic effect of OM19r and other aminoglycoside antibiotics (kanamycin, streptomycin, tobramycin, spectinomycin) was demonstrated, while no such effect was observed with other types of antibiotics (e.g., rifampicin, enrofloxacin) [136]. In summary, OM19r can be used together with aminoglycosides, contributing to the development of new methods of treating drug-resistant bacterial infections.

Another synthetic peptide based on oncocin is Oncocin-112 (Onc112), which has been modified to improve the efficacy of the parent peptide. In comparison with oncocin, the Onc112 sequence (VDKPPYLPRPRPPRrIYNr-NH2) contains D-Arg residues instead of L-Arg at positions 15 and 19 [76,77]. It was shown that these substitutions reduced the Onc112 susceptibility to proteases, which resulted in an increased half-life of the peptide in serum [77]. Studies on the structure of Onc112 showed that it can bind to the bacterial ribosome and form a complex with short mRNA and deacylated tRNA_fMet_ and tRNA_Met_ at the P-site, or it can enter the NPET and reach the PTC, where the aminoacyl-tRNA binding site is blocked. In addition, it has been shown that, when Onc112 enters ribosomes, it causes them to stop at the initiation site, blocking the elongation of translation [150,151,152]. In recent studies conducted by Zhu et al. [78], Onc112 was found to exert similar effects to those of the antibiotic kasugamycin. Onc112 is able to disrupt the normal growth and division of *E. coli* cells, which leads to a reduction in the length of bacterial cells. It also causes a change in the cellular location of ribosomes, DNA, and EF-Tu elongation factors [78].

Using different murine infection models, the in vivo efficacy of Onc112 and Onc72 (VDKPPYLPRPRPPROIYNO-NH_2_), an oncocin analog with less adverse effects in mice, was evaluated after intravenous or intraperitoneal administration in infected mice [153]. Both peptides reached the plasma, urine, kidney, liver, and brain within 10 min of treatment initiation. Onc112 provided a 100% survival rate in the *E. coli*-infected mice, while Onc72 was highly efficient in two lethal infection murine models in the treatment of *E. coli* and MDR carbapenemase-2-producing *K. pneumoniae* bacteremia. In addition, Onc72 administered to the mice 1h after the injection of a lethal dose of *E. coli* ATCC 25922 led to a significant reduction in the bacterial burden in the blood and to the recovery of all the animals [154]. Onc72 was also effective against *K. pneumoniae* ATCC 43816 when administered subcutaneously to neutropenic mice [155]. Recently, Onc72-based prodrugs in which Onc72 was coupled to 5 kDa and 20 kDa polyethylene glycol (PEG) were developed and their cytotoxic activity and pharmacokinetic parameters were tested in vitro and in vivo, respectively [156]. The prodrugs exhibited only slight cytotoxicity towards human cell lines (HEK293 and HepG2) and no hemolytic activity against human erythrocytes. It was demonstrated that, in the form of prodrugs, Onc72 is protected from proteolysis, allowing the extension of its half-life time, and the successive release ensured relatively constant concentrations of the peptide in serum. The Onc72 release and its extended half-life time in blood after subcutaneous administration was confirmed in female CD-1 mice, indicating the usefulness of this strategy to overcome the pharmacokinetic limitations of the PrAMP analogs [156].

### 5.3. ARV-1502 Peptide

The ARV-1502 peptide (Chex1-Arg20 amide) and its dimer A3-APO were designed based on the sequences of insect PrAMPs such as *P. apterus* pyrrhocoricin and *D. melanogaster* drosocin, with a main target being the DnaK chaperone. ARV-1502 is a non-membrane lytic peptide and can bind DnaK proteins from different bacterial species [157,158]. Binding to this chaperone occurs between the YLPRP motif of the peptide and the nucleotide-binding domain of DnaK and results in the inhibition of the DnaK-mediated phosphate release from the ATP. It also prevents substrate binding by the inhibition of multi-helical lid movement in the DnaK molecule. In the study conducted by Otvos et al. [159], ARV-1502 and the A3-APO dimer were tested for activity against drug-resistant Gram-negative bacteria. It was found that A3-APO in combination with the antibiotic colistin had a synergistic effect against *K. pneumoniae* and *A. baumannii*. Moreover, in studies on mice, a 100% survival rate was observed after the administration of A3-APO and colistin, compared to the administration of colistin alone (60% survival) [159]. On the other hand, the ARV-1502 monomer shows synergistic activity with another antibiotic, meropenem, against uropathogenic *E. coli* strains. The antibiotic increases bacterial membrane permeability, which consequently allows ARV-1502 to penetrate more easily into the bacterial cells. In addition, ARV-1502 binds the DnaK protein better than the dimer, which causes more effective disruption of the bacterial life processes. Hence, ARV-1502 and A3-APO may increase the effectiveness of antibiotics against drug-resistant pathogens [159]. Moreover, Xiong et al. [160] provided evidence of the superior therapeutic efficacy of ARV-1502 over imipenem/cilastatin in a mouse bacteremia model caused by MDR *A. baumannii* [160].

However, the YLPRP motif, important for ARV-1502 interaction with DnaK, has also been shown to be crucial for PrAMPs binding to the bacterial ribosome and for bacterial uptake [108]. Using a library of 176 ARV-1502 analogs, Brakel et al. [161] examined the effects of various substitutions in the D^3^KPRPYLPRP^12^ sequence on ribosome binding, the effectiveness of translation inhibition, and antibacterial activity. The YLPRP motif also plays a key role in the interaction of ARV-1502 with the 70S ribosome. The substitution of amino acid residues in this motif did not improve binding to the ribosome. On the contrary, the replacement of Tyr8 considerably reduced binding, whereas the substitution of Pro10 and Pro12 reduced binding to the ribosome and antibacterial activity. It was concluded that the sequence D^3^KPRPYLPRP^12^ enables the best ribosome binding of ARV-1502. Furthermore, it was shown that, with increasing peptide charge, the in vitro translation process is more strongly inhibited and the antibacterial activity against *E. coli* increases [161]. The binding site to the ribosome is similar for ARV-1502, pyrrhocoricin, and Onc112. Peptides of this type bind to the N-terminus in the NPET of the 50S subunit, which coincides with the A- and P-site of the ribosome. It was surprising to find that, despite the similar binding site of ARV-1502 and Onc112 to the ribosome, in both cases via the YLPRP motif, Onc112 completely inhibited the translation of the green fluorescence protein (GFP) in the cell-free assay, in contrast to ARV-1502. The ARV-1502 dimer, A3-APO, had this ability, however with an IC_50_ value still 10 times higher than Onc112. This was probably due to the twice as large positive charge of the dimer molecule. The strong electrostatic interactions between the positively charged peptide and the negative electrostatic potential of the NPET stop translation before the elongation step [161]. Interestingly, unlike other PrAMPs, ARV-1502 and A3-APO do not use typical transporters such as SbmA and MdtM for cellular uptake, but enter the bacterial cell via passive diffusion or use another transporter in an active mechanism. Passive transport may result from the substitution of several residues into Phe or Leu, which increases the hydrophobicity of the peptide molecule [161].

When developing new peptide analogs with a view to their potential use as future therapeutics, an important criterion of antimicrobial pharmacodynamics is the post-antibiotic effect (PAE) that is determined in vitro. It was observed that, after 1 h of exposure of *E. coli*, *K. pneumoniae*, and *P. aeruginosa* strains to A3-APO (and also Api88 and Api137), the PAEs were typically 4-fold stronger than those of the conventional antibiotics gentamicin and kanamycin [162]. In addition, the ability of PrAMPs to inactivate bacterial toxins (through the inhibition of their proper folding) is encouraging because these are frequently as lethal as bacteremia. This was shown for A3-APO, which restricted the proliferation of *B. anthracis* in infected isolated macrophages and was efficient in systemic mouse challenge models where A3-APO improved survival after intraperitoneal or intramuscular administration [163]. Moreover, A3-APO exhibited greater effectiveness than conventional antibiotics in mouse models of *S. aureus* blast wound infections, in mixed infections of foot ulcers by *K. pneumoniae*, *A. baumannii*, and *Proteus mirabilis*, and in *K. pneumoniae* lung infections mimicking ventilator-induced nosocomial infections. Considering A3-APO’s ability to deactivate bacterial toxins and to up-regulate anti-inflammatory cytokines (interleukins IL-4 and IL-10), it has been proposed that one of the modes of A3-APO action in vivo may be the inhibition of the inflammatory process induced by pathogens [164,165].

Li et al. [166] undertook the analysis of the antibacterial activity of the C-terminal hydrazide analog of the Chex1-Arg20 amide (ARV-1502) in the form of dimers prepared via bioconjugation with different linkers. They showed that tetrafluorobenzene and octofluorobiphenyl homodimers have greater antibacterial activity against *A. baumanii*, including the MDR strain FADDI-KP028 and the extensively drug-resistant (XDR) strain FADDI-AB156. The tested analogs caused the permeabilization of the inner and outer membrane of the bacteria, which led to membrane fragmentation and cell lysis. Importantly, these peptides caused a 50% reduction in preformed biofilms. In addition, they exhibited anti-inflammatory activity via the suppression of nitric oxide production in macrophages induced by *A. baumanii* LPS [166].

Although synthetic PrAMPs are considered mainly as molecules with activity against Gram-positive and Gram-negative bacteria, they also exhibit antifungal activity against *Cryptococcus neoformans*. Brakel et al. [167] analyzed the designed peptides Api88, Api137, Onc72, Onc112, and Chex1-Arg20 (ARV-1502) against different strains of *C. neoformans*, including *C. neoformans* 1841, H99, and KN99α, which dominate in different parts of the world. Chex1-Arg20, Api88, and Onc112 exhibited activity against *C. neoformans*, with Chex1-Arg20 having the highest activity. Experiments with 5(6)-carboxyfluorescein-labeled PrAMPs showed fluorescence in the cytoplasmic region of the fungal cells, but no binding of these peptides to the 80S ribosome was detected. However, the literature data indicate that another PrAMP, Bac7(1–35), inhibits translation in the eukaryotic system [168]. In comparison with the three analogs, Api137 had lower antifungal activity. On the other hand, natural PrAMPs, such as apidaecin 1B, drosocin, and pyrrhocoricin, did not show antifungal activity against *C. neoformans*. The differences could be related to the lower charge and less efficient cellular uptake compared to synthetic analogs. Chex1-Arg20, Api88, and Onc112 were detected in the cytoplasm of *C. neoformans* cells, i.e., they were able to overcome the barriers of the negatively charged capsule, cell wall, melanin layer, and cell membrane [167]. The amino acid sequences of the derivatives of selected natural PrAMPs are presented in Table 4.

Bacteriophage therapy may be a replacement therapy for antibiotics, but there are certain limitations to the use of this approach, as it is characterized by targeted action on a single bacterial strain and may lead to progressive resistance to phages. An example of such phage therapy is the experimental combination of lytic T7Select phages with synthetic apidaecin analogs, i.e., antibacterial Api802 and Api806, to effectively fight *E. coli* bacteria. In an experiment in which Api802 and Api806 were introduced into *E. coli* cultures previously infected with the T7Select phage, a beneficial effect on the control of the resistant bacterial strains was observed. At the same time, it was shown that an excess concentration of Api802 negatively influenced the efficacy of the T7Select phage, which may result in the inability of the phage to infect and kill bacteria. Moreover, appropriate amounts of these peptides were produced when plasmids encoding Api802, Api806, and Api810 sequences in *E. coli* were used. However, the integration of the apidaecin analogs coding sequences into the T7Select phage genome resulted in the production of these analogs being almost undetectable, and no effect of the peptides on the growth of phage-resistant bacterial strains was observed. Further research and analyses are needed to help improve these phages so that they can induce better apidaecin expression in bacterial cells and achieve the intended synergistic result [169].

### 5.4. New Strategies for Optimizing PrAMP Analogs

One of the great advantages of solid-phase peptide synthesis (SPPS) is its high efficiency, enabling the fast synthesis of different peptides, including peptide mixtures like peptide combinatorial libraries, from natural and non-natural building blocks, i.e., L- and D-amino acids. Moreover, by SPPS, stereorandomized (*sr*) peptides can be synthesized using racemic amino acids, giving an additional opportunity for the improvement of the properties of synthetic peptides and to explore the mechanisms of AMP action. Stereorandomized analogs of synthetic short α-helical AMPs (12 aa or 13 aa), as well as the random coil AMP indolicidin (13 aa), exhibited distinct properties in comparison with all L-peptides, all D-peptides, and racemic mixtures, in particular in terms of membrane disruptive activity, hemolytic activity, and cytotoxicity. Stereorandomization abolished the activity of α-helical AMPs but preserved the activity of random coil indolicidin. Importantly, *sr*-peptides were resistant to proteolytic degradation in serum [170]. In the optimization of the properties of the PrAMP oncocin, an approach of partial stereorandomization was used by Gan et al. [171]. It was demonstrated that this strategy protected the peptide sequence against proteolytic degradation in serum and was compatible with the peptide binding to its target. Oncocin, being a non-lytic PrAMP, acts intracellularly by binding to 70S ribosomes via its N-terminal region (14 residues), leading to the inhibition of translation in susceptible Gram-negative bacteria. While the D-Onc enantiomer and the full-sequence stereorandomized peptide *sr*-Onc lost the ability to bind to the ribosome, the *sr*9Cterm-Onc analog, in which up to nine C-terminal residues were stereorandomized, retained its ribosome binding ability and antibacterial activity against *E. coli* and *K. pneumoniae*. The research on different stereorandomized oncocin analogs has provided new insights into the mechanism of its action, as even those deprived of the ability of ribosome binding exhibited antibacterial activity in diluted growth media against *E. coli*, *K. pneumoniae*, *P. aeruginosa*, and *A. baumanii*. It is possible that, similarly to L-Onc, these conditions stimulated the uptake of the peptides [171]. This new approach paves the way to better understanding and improving the activities and properties of bioactive peptides.

In order to further expand the possibilities of analyzing structure–activity relationships and identifying potent AMPs, a high-throughput platform for the efficient sequence–activity mapping of AMPs via depletion (SAMP-Dep) has recently been developed by DeJong et al. [172]. The AMP mutant library prepared in a proper expression vector is used for a bacterial host culture transformation, and the intracellular expression of AMPs is induced. The host bacterial cells are grown under selective pressure and are subjected to deep sequencing for mutant frequency quantification. Using the SAMP-Dep platform, the intracellular activity of 170,000 oncocin variants against *E. coli* was analyzed. Based on the obtained results, synthetic first- and second-generation peptide libraries were designed, and finally a variant with two-fold increased intra- and extracellular activity was successfully selected and identified [172]. The SAMP-Dep platform has recently been used to analyze other invertebrate PrAMPs, namely metalnikowin (Met) and apidaecin-1B (Api), representing Class I and Class II PrAMPs, respectively. In this study, 26,000 Met variants and 32,000 Api variants were characterized in terms of the structure–activity landscape. It was found that mutations that were most beneficial for their activity were present within the C-terminal and N-terminal region of Met and Api, respectively. A proline mutation within the PRP motifs eliminated the activity, whereas non-PRP prolines tolerated the mutation. The synthesized selected Met and Api variants exhibited increased activity or specificity against different strains of *E. coli* and *S. enterica* [173].

In another study, single-amino-acid substitutions were introduced in the Api sequence in order to map the entire sequence space available for altering and possibly improving Api antimicrobial properties [174]. A comprehensive library of *api* gene variants with all possible single-amino-acid substitutions was obtained and expressed endogenously in *E. coli* cells. It was demonstrated that the Api variants expressed in vivo affected bacterial cell growth. To determine the sequence–activity relationships, an analysis of the library by next-generation sequencing was conducted. Such an approach allowed the identification of the on-target activity (inhibition of translation by binding to the ribosome) of every single-amino-acid-substituted Api variant in a sole experiment. Furthermore, the identification of inactivating mutations enabled defining the pharmacophore (five C-terminal residues) in the Api sequence [174].

The studies presented above show the undoubted advantages of using gene libraries, including combinatorial libraries, and the use of intracellular expression in bacterial cells to identify new, more effective variants of known PrAMPs targeting ribosomes or other intracellular targets. Moreover, these high-throughput strategies provide broad and detailed information on the importance of individual amino acid residues for the interaction of the peptide with its intracellular target. However, despite these advantages, it should be noted that the in vivo expression of AMPs directly in the bacterial host cell bypasses the step of peptide entry into the cell. The better binding of peptides selected using these strategies to the intracellular target and their higher antimicrobial activity do not guarantee satisfactory cellular uptake, resistance to proteases, or stability in salt solutions. Regardless of binding to the target and the inhibition of processes inside the bacterial cell, whether the selected peptides meet the remaining criteria, including a lack of cytotoxic and hemolytic activity, should be tested.

This issue was also addressed and discussed in a work on ARV-1502 analogs reported by Brakel et al. [175]. In this study, ARV-1502 analogs were designed by substituting residues important for anti-Gram-negative bacteria activity, synthesized using SPPS, and their binding to and influence on the refolding and ATPase activity of DnaK/DnaJ/GrpE chaperone systems from Gram-negative *E. coli* and Gram-positive *S. aureus* was examined. Of the 183 analogs tested, 15 analogs with increased hydrophobicity exhibited better binding to at least one of the DnaK proteins. The interaction of these hydrophobic analogs with *E. coli* chaperones resulted in a considerable reduction in chaperone refolding activity, while ATPase activity was hardly affected. However, peptides bearing aspartate substitutions caused a decrease in ATPase activity in the *E. coli* chaperones, whereas an increase in this activity in the *S. aureus* chaperones was observed. This was also reflected in the increased activity against *S. aureus*, compared to the starting peptide AVR-1502. It was shown that substitutions with Asp and Ser were unfavorable for the action against *E. coli* but significantly improved anti-*S. aureus* activity, suggesting the altered cellular uptake of these AVR-1502 analogs [175]. This work demonstrates that research on PrAMP analogs can lead to the development of PrAMP-derived peptides that are not only more effective against Gram-negative pathogens, but also exhibit activity against Gram-positive pathogenic bacteria.

## 6. Conclusions

The diversity of PrAMPs naturally occurring in invertebrate animals concerns, among others, their molecular structure. This diversity can be a result of only a few mutations in some peptides, as in the case of the apidaecin homologs identified recently in bees and wasps. It reflects the adaptability of the hosts to the spectrum of insect pathogens and shows that minor alterations differentially affect the activity of these PrAMPs against various pathogens. Other invertebrate PrAMPs, in addition to the proline-rich domain, contain domains of other types, e.g., a cysteine-rich domain or a whey acidic protein domain. Although not all of these peptides meet the recently postulated criteria for PrAMPs, their proline-rich domains confer their specific properties, which, in combination with other domains present in the molecule, translate into diverse biological activities. Such peptides may constitute a starting point and offer new possibilities for the development of new bioactive molecules.

Modern computational approaches using different prediction algorithms, in silico models, and molecular dynamics simulations in alignment with selection from peptide combinatorial libraries, the use of directed evolution strategies, and advanced peptide microarrays provide increasingly better tools for the development and optimization of novel AMPs with desired properties. Moreover, the drug-like properties of natural and optimized AMPs can be further predicted, including chemical absorption, distribution, metabolism, excretion, and toxicity (ADMET), i.e., features that are essential in the development of new high-quality drug candidates. For example, promising drug-like properties of seven AMPs (including abaecin and apidaecin) have recently been demonstrated using the ADMETlab, OECD QSAR toolbox, and VEGA HUB virtual environments, confirming their suitability as potential leads for the pharmaceutical and food industries [176]. However, for all these activities to bring the desired result in the form of new effective and safe therapeutics, detailed studies of the mechanisms of their action at the molecular level and research into the potential mechanisms of pathogen resistance should be continued.

## Figures and Tables

**Table 1:** Domain structure of selected invertebrate PrAMPs.

Class	Peptide Name	Variant	Structural Domains
*Crustacea*	panaeidins	PEN3	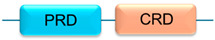
	crustins	Type III	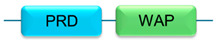
	stylicins	1	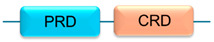
	arasins	Arasin 1	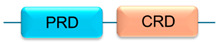
*Mollusca*	*Cg*-Prp		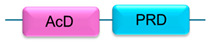
*Insecta*	drosocin metchnikowin pyrrhocoricin oncocin apidaecins abaecins lebocins Pro1, Pro2		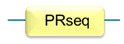
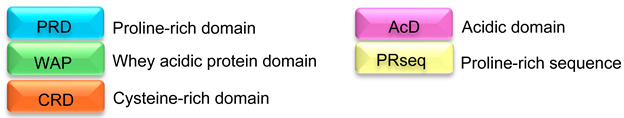			

**Table 3:** Properties of selected natural insect PrAMPs. Pro-Arg-Pro (PRP) motifs, glycosylated Thr (T) and Ser (S) residues, and Pro residues outside the PRP motifs are marked in blue, red, and bold, respectively.

Order	Species	Peptide Name	Sequence	aa	% P	Ref.
Diptera	*Drosophila melanogaster*	Drosocin	GKPRPY**S**PRP**T**SHPRPIRV	19	32	[64]
		Metchnikowin	HRHQG**P**IFDTR**P**S**P**FN**P**NQPRPGPIY	26	23	[50]
	*Lucilia* *sericata*	LSer-PRP1	EWR**P**HGSNGGSSLR**P**GR**P**QTL**PP**QR**P**IQ**P**DFNG**P**RQRF	38	21	[59]
		LSer-PRP2	EWR**P**HGSIGGSGLR**P**GR**P**QTL**PP**QRPRR**P**DFNG**P**RHRF	38	21	
		LSer-PRP3	S**P**FVDR**P**RR**P**IQHNG**P**K**P**RIITN**PP**FN**P**NAR**P**AW	34	26	
		LSer-PRP4	SWIKKDKF**P**SSTG**P**YN**P**N**PPPP**RF	24	29	
Hemiptera	*Pyrrhocoris apterus*	Pyrrhocoricin	VDKGSYLPRP**T****P**PRPIYNRN	20	25	[65]
	*Palomena prasina*	Metalnikowin-I	VDK**P**DYRPRPRP**P**NM	15	33	[66]
	*Oncopeltus fasciatus*	*Oncopeltus* antibacterial peptide 4	VDK**PP**YLPRP**PPP**RRIYNNR	20	35	[67]
Hymenoptera	*Apis* *mellifera*	Abaecin	YV**P**L**P**NV**P**Q**P**GRR**P**F**P**TF**P**GQG**P**FN**P**KIKW**P**QGY	34	29	[68]
		Apidaecin	GNNR**P**VYISQPRP**P**H**P**RL	18	28	[69]
		Apidaecin 1a	GNNR**P**VYI**P**QPRP**P**H**P**RI	18	33	
		Apidaecin 1b	GNNR**P**VYI**P**QPRP**P**H**P**RL	18	33	
		Apidaecin-2	GNNR**P**IYI**P**QPRP**P**H**P**RL	18	33	
	*Bombus pascuorum*	Abaecin	FV**P**YN**P**PRPGQSK**P**F**P**SF**P**GHG**P**FN**P**KIQW**P**Y**P**L**P**N**P**GH	39	33	[70]
		Apidaecin	GNR**P**VYI**PP**PRP**P**H**P**RL	17	41	
	*Myrmecia* *gulosa*	Formeacin-1	GR**P**N**P**VNNK**P****T****P**H**P**RL	16	31	[71]
		Formeacin-2	GR**P**N**P**VNTK**P****T****P**Y**P**RL	16	31	
Lepidoptera	*Bombyx* *mori*	Lebocin 1/2	DLRFLY**P**RGKL**P**V**P****T****PPP**FN**P**K**P**IYIDMGNRY	32	25	[72]
		Lebocin 3	DLRFLY**P**RGKL**P**V**P****T**L**PP**FN**P**K**P**IYIDMGNRY	32	22	
		Lebocin 4	DLRFWN**P**REKL**P**L**P**TL**PP**FN**P**K**P**IYIDMGNRY	32	22	
	*Galleria mellonella*	Proline-rich peptide 1	DIQI**P**GIKK**P**THRDIII**P**NWN**P**NVRTQ**P**WQRFGGNKS	37	14	[73]
		Proline-rich peptide 2	EIRL**P**E**P**FRF**P**S**P**TV**P**K**P**IDID**P**IL**P**H**P**WS**P**RQTY**P**IIARRS	42	26	
	*Heliothis virescens*	Heliocin	QRFIH**P****T**YR**PPP**Q**P**RR**P**VIMRA	22	27	[8]

**Table 4:** Selected synthetic analogs developed based on natural invertebrate PrAMPs.

Peptide Name	Sequence	Reference
Api137	gu-ONNRPVYIPRPRPPHPRL-OH	[146]
Api88	gu-ONNRPVYIPRPRPPHPRL-NH_2_	[139]
Api805	GNNRPIYIPRPRPPHPRPIRV-OH	[135]
OM19r	VDKPPYLPRPRPIRrPGGr-NH_2_	[136]
Oncocin-112	VDKPPYLPRPRPPRrIYNr-NH_2_	[78]
Oncocin-72	VDKPPYLPRPRPPROIYNO-NH_2_	[153]
ARV-1502	Chex-RPDKPRPYLPRPRPPRPVR-NH_2_	[161]

## Data Availability

Data available on request from the authors.
